# A multiscale model to predict current absolute risk of femoral fracture in a postmenopausal population

**DOI:** 10.1007/s10237-018-1081-0

**Published:** 2018-10-01

**Authors:** Pinaki Bhattacharya, Zainab Altai, Muhammad Qasim, Marco Viceconti

**Affiliations:** 1 Department of Mechanical Engineering, University of Sheffield, The Sir Frederick Mappin Building, Mappin Street, Sheffield, S1 3JD UK; 2 INSIGNEO Institute for in Silico Medicine, University of Sheffield, The Pam Liversidge Building, Mappin Street, Sheffield, S1 3JD UK

**Keywords:** Osteoporotic hip fracture, Multiscale model, Verification, Uncertainty quantification, Validation

## Abstract

Osteoporotic hip fractures are a major healthcare problem. Fall severity and bone strength are important risk factors of hip fracture. This study aims to obtain a mechanistic explanation for fracture risk in dependence of these risk factors. A novel modelling approach is developed that combines models at different scales to overcome the challenge of a large space–time domain of interest and considers the variability of impact forces between potential falls in a subject. The multiscale model and its component models are verified with respect to numerical approximations made therein, the propagation of measurement uncertainties of model inputs is quantified, and model predictions are validated against experimental and clinical data. The main results are model predicted absolute risk of current fracture (*ARF0*) that ranged from 1.93 to 81.6% (median 36.1%) for subjects in a retrospective cohort of 98 postmenopausal British women (49 fracture cases and 49 controls); *ARF0* was computed up to a precision of 1.92 percentage points (pp) due to numerical approximations made in the model; *ARF0* possessed an uncertainty of 4.00 pp due to uncertainties in measuring model inputs; *ARF0* classified observed fracture status in the above cohort with AUC = 0.852 (95% CI 0.753–0.918), 77.6% specificity (95% CI 63.4–86.5%) and 81.6% sensitivity (95% CI 68.3–91.1%). These results demonstrate that *ARF0* can be computed using the model with sufficient precision to distinguish between subjects and that the novel mechanism of fracture risk determination based on fall dynamics, hip impact and bone strength can be considered validated.

## Introduction

By 2020, in the UK, the annual cost of hip fracture treatment will exceed £2 billion with over 100,000 new hip fractures per year (Burge et al. 2001). Hip fractures are associated with excess mortality that lasts up to several years after the surgery required to stabilize the fracture (Abrahamsen et al. 2009). Thus, the prevention of osteoporotic hip fractures is a high-priority healthcare problem. In designing effective strategies for hip fracture prevention, a key question has remained unanswered: which specific factors most strongly determine fracture risk?

A person’s risk of hip fracture is dependent on a several factors (Cummings et al. 1995). Frequency of falling is a known risk factor, with 73–83% of hip fractures in elderly women resulting from a fall (Costa et al. 2013). Fall severity also independently influences fracture risk, which explains why only 3% of falls result in a hip fracture (Greenspan et al. 1994; NICE 2013). Fracture risk depends on bone strength as well, which is the minimum load required to fracture a bone from a given impact orientation. In the current standard-of-care for predicting fracture risk using FRAX™ (Kanis et al. 2009), dual-energy X-ray absorptiometry scan-based areal bone mineral density (DXA-aBMD) measured at the femoral neck is used as a surrogate measure of bone strength. Ageing is another risk factor, as it leads to progressive losses in bone strength (Paggiosi et al. 2011) and in neuro-motor control (Larsson and Ramamurthy 2000) which can cause fall frequency to increase. Ensrud (2013) recently described in detail the epidemiology of hip fracture risk with advancing age. Currently, significant challenges exist in developing mechanistic models that capture the role of ageing and can accurately predict hip fracture risk (Christen et al. 2010). Statistical regression models such as FRAX™ account for ageing by considering age as a determinant of 10-year fracture risk (Kanis et al. 2008).

This paper focusses on the current absolute hip fracture risk *ARF0*, which is defined as the risk of sustaining a fracture over a period just short of a year. Ageing-related changes are measurable only over periods spanning several years because these are controlled by processes at the cellular level such as bone remodelling (Raggatt and Partridge 2010) and muscle hypotrophy (Larsson and Ansved 1995). Therefore, in quantifying *ARF0*, the effect of ageing may be neglected, and only the risk factors associated with fall severity and bone strength need to be accounted for.

Mechanistic models are well suited to analyse the dependence of fracture risk to different risk factors, and especially for ranges of risk factor values for which observational data is not available. The aim of this study is to validate a mechanistic model to predict *ARF0* that accounts for whole-body dynamics during a fall, hip impact with the ground following the fall and femur strength loaded in a side-fall configuration. Validity of the *ARF0* model is quantified by its accuracy in classifying hip fracture status in a retrospective cohort of postmenopausal elderly British women. Once validated, the model will allow one to quantify how fracture risk (*ARF0*) changes when parameters corresponding to fall dynamics, hip impact and side-fall strength are modified by one or more risk factors.

Validation of a mechanistic model for hip fracture risk has been identified as a grand challenge (Christen et al. 2010; Viceconti et al. 2008). This is because the variables that determine fall dynamics, hip impact and side-fall strength (and thus quantify the risk factors) occupy a large space–time domain. Experimental measurement of bone strength requires features down to 10^−4^ s to be captured (Schileo et al. 2014), while experimental measurement of whole-body dynamics variables—which determine fall severity—require observation periods of ~ 10^3^ s (Terrier and Reynard 2015). There is no experimental modality that spans the entire domain from 10^−4^ to 10^3^ s. With current computing capabilities, modelling this domain accurately is also prohibitive.

To overcome this challenge, a multiscale modelling approach is used in this study to compute *ARF0*. Component models are developed to predict peak fall impact force, force-transfer between the ground and skeleton at the point of impact and bone strength under side-fall loading configuration. These models correspond to whole-body dynamics, hip impact and femur fracture experiments. The component models are coupled to form the multiscale model.

The present approach to compute *ARF0* is novel in several aspects compared to previous approaches to mechanistic multiscale modelling of hip fracture (Bouxsein et al. 2007; Dufour et al. 2012; Sarvi and Luo 2015). First, the full range of potential impact force magnitudes and orientations to which a subject may be exposed are considered. This approach differs from previous studies where fractures were considered to occur only under one specific fall scenario (Bouxsein et al. 2007; Sarvi and Luo 2015). It allows the analysis of sensitivity of *ARF0* to changes in distribution of impact force magnitude and/or orientation, which can capture the effect of a fracture risk reduction intervention.

Second, bone strength is determined using a computed-tomography (CT)-based finite-element (FE) modelling pipeline. The CT-FE method predicts bone strength more accurately than DXA-based FE models or DXA-aBMD-based statistical models used in previous work (Bouxsein et al. 2007; Dall’Ara et al. 2016; Falcinelli et al. 2014; Sarvi and Luo 2015; Viceconti et al. 2018). The accuracy of the CT-FE pipeline used in the current work has been detailed elsewhere (Schileo et al. 2014; Viceconti et al. 2018) and is comparable to other similar approaches reported in literature (Bessho et al. 2004, 2007, 2009; Keyak 2001; Keyak et al. 2005; Keyak and Rossi 2000; Keyak et al. 1997, 2011; Nishiyama et al. 2014). Specifically, failure strength and strains in cadaver bones are predicted using this CT-FE pipeline with 15% and 7% standard error of estimate, respectively. Accuracy in predicting bone strength underpins the accuracy of fracture prediction in live subjects using this CT-FE pipeline, as reported in Falcinelli et al. (2014), Qasim et al. (2016) and Viceconti et al. (2018). This accuracy is similar to other fracture prediction models that also use CT-FE (Adams et al. 2018; Keyak et al. 2011; Panyasantisuk et al. 2018; Qasim et al. 2016). Yet, it must be noted that fracture risk prediction based on bone quality only cannot provide insight into the role of fall mechanics on fracture risk, which is the key objective of the present paper. Thus, a comprehensive review of the prediction of fracture risk based on bone strength is not attempted, and we point the interested reader to a recent exposition by Viceconti et al. (2018).

Third, *ARF0* is defined as a purely frequentist probability measure and can therefore be compared directly to an observable risk quantity such as *ARF10* (Siris and Delmas 2008). This was not possible in past studies (Bouxsein et al. 2007; Sarvi and Luo 2015) where the ratio of a single-valued fall force to a single-valued bone strength was used. The frequentist approach can also naturally account for a variable fall rate.

Finally, the accuracy of prediction is evaluated in terms of classification of observed fracture status, thus fulfilling a stricter requirement than association reported in past work (Bouxsein et al. 2007; Dufour et al. 2012; Sarvi and Luo 2015). To the best of our knowledge, similar probabilistic modelling approaches have recently gained attention in the prediction of hip fracture risk (Jiang et al. 2015; Viceconti et al. 2012). In these studies, the deterministic prediction of bone strength based on FE modelling was augmented by applying a statistical distribution of loads accounting for the variability of falls. Similar to the present study, Viceconti et al. (2012) investigated the sensitivity of fracture risk to various factors, but did not validate the predicted fracture risk in the sense of association with or classification of observed fracture status. Jiang et al. (2015) solved an optimization problem for classification accuracy, but in doing so, obviated the possibility of independently validating the choice of loading distribution. The FE model used by Jiang et al. (2015) was not validated against cadaver experiments unlike the FE model used in this paper.

The following sections detail the multiscale model for *ARF0* along with the component models comprising it, and the results of verification, uncertainty quantification and validation analyses of all models.

## Materials and methods

The multiscale model used to calculate *ARF0* comprises three component models: a model at the whole-body scale that predicts the impact force on the body applied by the floor during a fall; a model situated between the body and the organ (bone) scales that predicts the fraction of impact force transferred to the skeleton; and an organ-scale FE model that predicts bone strength.

The orchestration of these three models is considered a multiscale model because the three models are defined and identified at three different space–time scales, although partially overlapping. The model at the whole-body scale possesses, to use the terminology proposed in Bhattacharya and Viceconti (2017), an extent of 10^1^ m (distance covered in treadmill tests) and a grain of 10^−2^ m (spatial resolution in treadmill tests) over space, and an extent of 10^3^ s (duration of treadmill tests) and a grain of 10^−2^ s (temporal resolution in treadmill tests) over time. The organ-scale model has an extent of 10^0^ m (dimension of femur fracture test apparatus) and grain of 10^−3^ m (strain gauge resolution in femur fracture tests) over space, and an extent of 10^0^ s (duration of fracture tests) and a grain of 10^−4^ s (temporal resolution of fracture tests) over time. The body–organ relation model has a scale somehow intermediate to these two.

All three component models are detailed below, followed by a description of the multiscale model. A list of all abbreviations and symbols used is provided in Table 1.

**Table 1:** List of abbreviations and symbols in their order of usage in the text

Abbreviation/symbol	Meaning
*ARF0*	Current absolute risk of hip fracture
*m*	Whole-body mass
COM	Centre of mass
*h*	Elevation of whole body COM from the ground when standing in an upright position
*H*	Whole body height
*c*	Ratio of COM elevation to whole body height
Bx, By, Bz	Body coordinate system with origin at COM
Gx, Gy, Gz	Ground coordinate system with origin at hinge
Fx, Fy, Fz	Femur coordinate system with origin at femoral head centre
*ψ*	Angle between Bx and fall plane (plane containing Gz and Bz)
*θ*	Angle between the vertical axis (Gz) and the line joining COM and hinge (Bz)
*θ*_*i*_	Value of *θ* when fall initiates
*θ*_*f*_	Value of *θ* when fall completes (impact)
*α*	Hip abduction angle at impact
*β*	Internal hip rotation angle at impact
$\dot{\theta }_{i}$	Rate of change of *θ* with respect to time when fall initiates
$\ddot{\theta }_{i}$	Second-derivative of *θ* with respect to time when fall initiates
*e*	Kinetic energy per unit body mass at impact
*g*	Acceleration due to gravity
*u*	Velocity of impact at the hip
*η*_*P*_	Impact energy attenuation due to postural defence
F*	Unattenuated impact force
*k*	Factor of proportionality
*∆t*	Duration of impact at the hip
*F*	Attenuated impact force
*η*_*I*_	Impact force attenuation due to all factors except passive trochanteric soft-tissues
*η*_*ST*_	Impact force attenuation due to passive trochanteric soft-tissues
*η*_*I*_^floor^	Impact force attenuation due to flooring
*η*_*I*_^ext^	Impact force attenuation due to hip protectors
*η*_*I*_^act^	Impact force attenuation due to active trochanteric soft-tissues
*BMI*	Body mass index
*S*	Femur strength
*G*	Discretized geometry of proximal femur
*E*	Discretized elasticity of proximal femur
*α*′	Angle between Fy and direction of impact force projected on Fy–Fz plane
*β*′	Angle between Fy and direction of impact force projected on Fx–Fy plane
*χ*	Fracture outcome linked to a fall
$p^{x}$	Probability density function for variable *x*
*P*	Probability that a fall will lead to a fracture
*N*	Sample-size of Monte–Carlo simulation
*n*	Annual fall rate
*a*	Lower truncation limit
*b*	Upper truncation limit
*STT*	Trochanteric soft tissue thickness
*μ*, μ*	Mean of truncated normal distribution
*σ* ^2^	Variance of truncated normal distribution
*ε* ^*μ*^	Errors in mean estimated from Monte–Carlo sample
*ε* ^*σ*^	Errors in variance estimated from Monte–Carlo sample
*ε* ^*ARF0*^	Error in estimating *ARF0* of a subject from a Monte–Carlo sample of falls
*S*_*i*_	First-order sensitivity index
*T*_*i*_	Total sensitivity index
*p*	Level of statistical significance
*r*	Correlation coefficient
AUC	Area under the Receiver Operating Characteristic curve

### Body–floor impact model

The body–floor impact model at the whole-body scale determines the magnitude of impact force due to a fall. Here, a fall is idealized as a rotation of the whole body on any plane containing the vertical axis (fall plane). The rotation occurs around a spherical joint (hinge) fixed to the floor and located near the foot on the side of impact. Factors which may reduce the impact, such as knee flexion or partial interruption of the fall, are not modelled dynamically, but accounted for empirically.

**Fig. 1:**
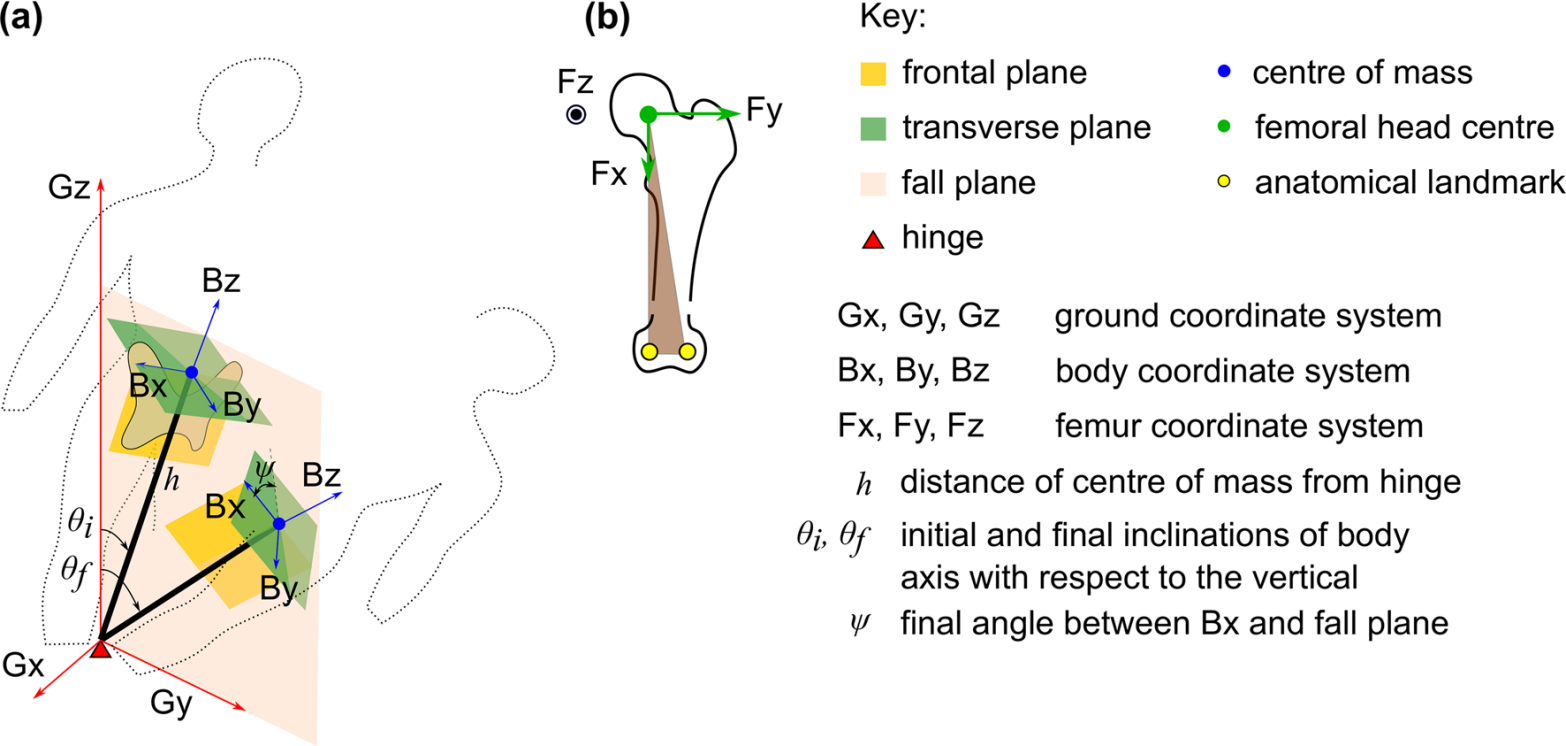
**a** The inverted pendulum abstraction of the body during a fall. Coordinate systems for the ground and the body are shown in red and blue arrows, respectively. The origins of the ground and body coordinate systems are identified with the hinge and the body centre of mass, respectively. **b** The femoral coordinate system (green arrows), with origin located at the centre of the femoral head. Fz points in the direction out of the plane of the paper

The model considers the body mass (*m*) to be concentrated at the moving end of an inverted pendulum, the static end of which is located at the hinge (Fig. 1). The pendulum length (*h*) equals the body centre-of-mass (COM) elevation from the ground in the upright position. It is taken to be a fixed proportion (*c* = 0.554) of the subject’s standing height (*H*) (Croskey et al. 1922). This description is underpinned by the fact that the instantaneous centre of rotation of the COM remains close to the ground during level walk (Herr and Popovic 2008).

In this model, the body coordinate axis system (Bx, By, Bz) originates at the COM with axes perpendicular to anatomical planes (Fig. 1a). The relative orientation of the body with respect to the ground (G) is given by angles *ψ* and *θ*, where *θ* (measured from Gz = vertical in the fall plane) varies from *θ*_*i*_ to *θ*_*f*_. Figure 1b shows the femoral coordinate axis system (Fx, Fy, Fz), with the centre of the femoral head as the origin and with respect to two other femoral anatomical landmarks. At the instant of impact, the orientation of the femur in relation to the body is specified by the angles *α* and *β*, which are the rotations of the femur axis perpendicular to the frontal and transverse planes of the body, respectively. The angles *α* and *β* are commonly known as hip abduction and internal hip rotation, respectively.

In these calculations, a fall is considered to ‘end’ at the moment the hip impacts the floor. For an inverted pendulum that initially possesses angular velocity $\dot{\theta }_{i}$ and angular acceleration $\ddot{\theta }_{i}$, the conservation of energy principle implies that the total kinetic energy per unit body mass available at the end of the fall is

1 $$ e = c^{2} H^{2} \ddot{\theta }_{i} \left( {\theta_{f} - \theta_{i} } \right) + \frac{1}{2}c^{2} H^{2} \dot{\theta }_{i}^{2} + gcH\left( {\cos \theta_{i} - \cos \theta_{f} } \right) $$

The rotation of joints of the lower limbs during fall causes work to be done by the associated muscles. This work reduces the kinetic energy available at the end of the fall compared with the COM remaining at a fixed distance from the hinge, as is assumed in Eq. (1). Reduction in kinetic energy at the end of the fall is also possible if there is partial interruption, such as by impact with other anatomical sites during the fall. Such reductions are termed here as postural reflex attenuation. Thus, the velocity of hip impact (*u*) is given by:

2 $$ u = \sqrt {2\left( {1  {-}  \eta_{\text{P}} } \right)e} $$

where *η*_P_ is the postural reflex attenuation coefficient. Dimensional considerations and experiments show that the peak impact force applied on the femur depends linearly on *u* and *m* (Laing and Robinovitch 2009; Robinovitch et al. 1991). Impact tests using a synthetic pelvis and a rigid floor (Laing and Robinovitch 2008, 2009; Robinovitch et al. 1995) show that impact force is a triangular function of time, reaching a peak at the middle of the total impact duration *∆t*. Hence, the peak impact force is modelled as:

3 $$ F^{*} = k \cdot 2mu/\Delta t.$$

Here, $k$ is a factor of proportionality accounting for the complexity of the interaction that is not modelled explicitly. The average experimental values *m* = 61.2 kg, *u* = 3 m/s, *∆t* = 0.09 s and F* = 2.05 kN (Laing and Robinovitch 2008, see Fig. 4b in their paper) suggest *k* ~ 0.5. This results in the simplification

4 $$ F^{*} = mu/\Delta t. $$

### Ground–skeleton force-transfer model

The body–organ relation model is henceforth referred to as the ground–skeleton force-transfer model. It predicts the fraction of peak impact force *F* transferred to the skeleton. The total peak impact force F* determined above considers the body to possess average passive soft tissue damping properties and to impact a rigid floor without a hip protector. In reality, the presence of various damping effects mean that the peak impact force will only be partially transferred to the skeleton. In the ground–skeleton force-transfer model, such effects are lumped into two force attenuation coefficients. The first coefficient (*η*_*I*_) accounts for damping due to flooring elements (i.e. carpets) (Laing and Robinovitch 2009), hip protector devices (if present) (Laing and Robinovitch 2008), and all active soft tissues (muscles) that contract at the instant of impact (Robinovitch et al. 1991). The second coefficient (*η*_*ST*_) accounts for damping due to all passive soft tissues interposed between the point of impact on the skin and the lateral aspect of the greater trochanter (which includes also the passive component of the muscular tissues) (Robinovitch et al. 1995). Thus, the attenuated peak impact force magnitude applied to the greater trochanter is:

5 $$ F = \left( {1 - \eta_{I} } \right)\left( {1 - \eta_{ST} } \right)F^{*} = \left( {1 - \eta_{I} } \right)\left( {1 - \eta_{ST} } \right)mu/\Delta t $$

The factor (1 − *η*_*I*_) is composed as the product (1 − *η*_*I*_^floor^)(1 − *η*_*I*_^ext^)(1 − *η*_*I*_^act^) where *η*_*I*_^floor^, *η*_*I*_^ext^ and *η*_*I*_^act^ are attenuation coefficients due to floor material, hip protectors and active soft tissue damping, respectively. The coefficient *η*_*ST*_ is considered to be a function of the body mass index BMI = *m*/*H*^2^. All attenuation coefficients are defined relative to the synthetic hip, rigid floor and no hip protector impact scenario (Laing and Robinovitch 2008).

### Femur strength model

The femur strength model at the organ scale determines the strength (*S*) of the femur given a fall loading direction. The three-dimensional bone geometry is discretized with 10-noded quadratic tetrahedral elements with a typical edge length of 3 mm; the discretization is referred to by the function *G*. Linear elastic isotropic properties are specified element-wise; this spatial heterogeneity is referred to by the function *E*.

Compared to other similar models, the CT-based subject-specific finite model used here relies on two major simplifications: local isotropy and fragile failure. The subject-specific modelling method we used captures the bone tissue heterogeneity with spatial resolution of around 2–3 mm (average finite element size); by assigning a different elastic module to each finite element based on the local mineral density, the model captures the spatial anisotropy at this characteristic length scale. Of course, bone is anisotropic also at much smaller scales, which one should homogenize into an anisotropic constitutive equation within each finite element. However, the improved accuracy of this refinement is mostly wasted by the fact we do not have reliable subject-specific measurements of such small-scale anisotropy. Since the modelling method we use, which account only for long-range anisotropy, was found to predict measured principal strains with a root-mean-squared error less than 7.2% (Schileo et al. 2008), we believe this simplification is acceptable. While in general bones fail with significant post-elastic work, in the particular case of proximal femur fractures produced under side-fall conditions all *ex vivo* experiment show that initial cracks fully propagate within a few milliseconds and without showing any appreciable post-elastic work. Thus, is this particular case a fragile failure criterion (maximum strain) is perfectly suitable, as confirmed by the excellent predictive accuracy of this model when compared to cadaveric strength measurements (Schileo et al. 2014).

**Fig. 2:**
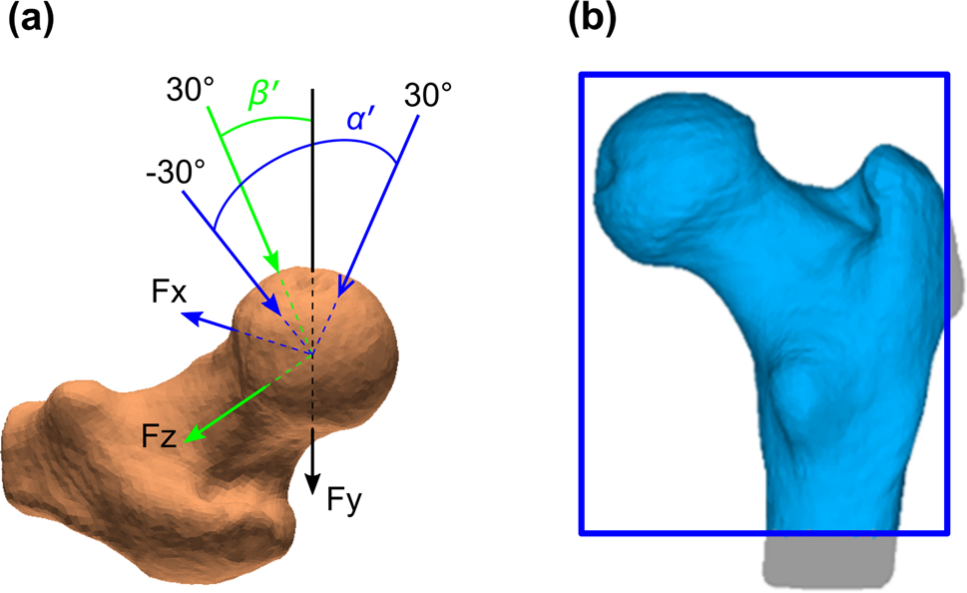
**a** In the fall configuration, a concentrated force is applied at the femoral head centre and in a direction specified by the angles *α′* and *β′* measured with respect to the femoral axes Fz and Fx, respectively. **b** The surface shown in blue is the region of interest (ROI) where the strain-based fracture criteria are evaluated. The surface outside the ROI, in grey, contains nodes where the solution is judged to be affected either by contact interaction (on the right) or by boundary constraints (at the bottom)

Briefly, loading in the fall configuration implies: (1) a concentrated force is applied at a node at the centre of the femoral head and in a direction specified by rotations *α*′ and *β*′ (Fig. 2a) taken, respectively, about the Fz and Fx axes; (2) hard, frictionless contact interaction is defined between the greater trochanter surface of the femur and a rigid static plane that is oriented normally to the direction of force; (3) nodes at the distal end of the proximal femur model are suitably constrained to remove any artificial motion arising from numerical discretization. For any pair of (*α*′, *β*′), the strength *S* is defined as the smallest magnitude of force required to cause the maximum principal strain to exceed + 0.73% or the minimum principal strain to fall below – 1.04% anywhere in a region of interest (ROI) (Fig. 2b) where the near totality of these low-energy impact fractures is initiated (Bayraktar et al. 2004; Qasim et al. 2016).

### Multiscale model for ARF0

The current absolute risk of fracture *ARF0* is defined as the probability that the subject will suffer a fracture in the period of under a year. The qualifier ‘current’ distinguishes *ARF0* from the more clinically relevant quantity *ARF10*, which is the risk of sustaining a fracture over a 10-year period (Siris and Delmas 2008). *ARF0* is computed as the probability that at least one out of *n* mutually independent falls will lead to a fracture, where *n* is the fall rate (in falls per person per year). Thus, if *P* is the probability that a random fall will lead to a fracture, then

6 $$ ARF0 = 1 \, {-}(1 \, {-}P)^{n} $$

following the binomial theorem. Note that *ARF0* ≤ 1 = 100%. *ARF0* is expressed henceforth in percentage units (%). The difference between any two *ARF0* values is expressed as percentage points (pp).

The probability *P* that a random fall will lead to a fracture is determined in two steps. In the first step, the fracture outcome of a specific fall is determined. In the second step, *P* is determined by accounting for the variability of fracture outcomes over a distribution of falls.

**Fig. 3:**
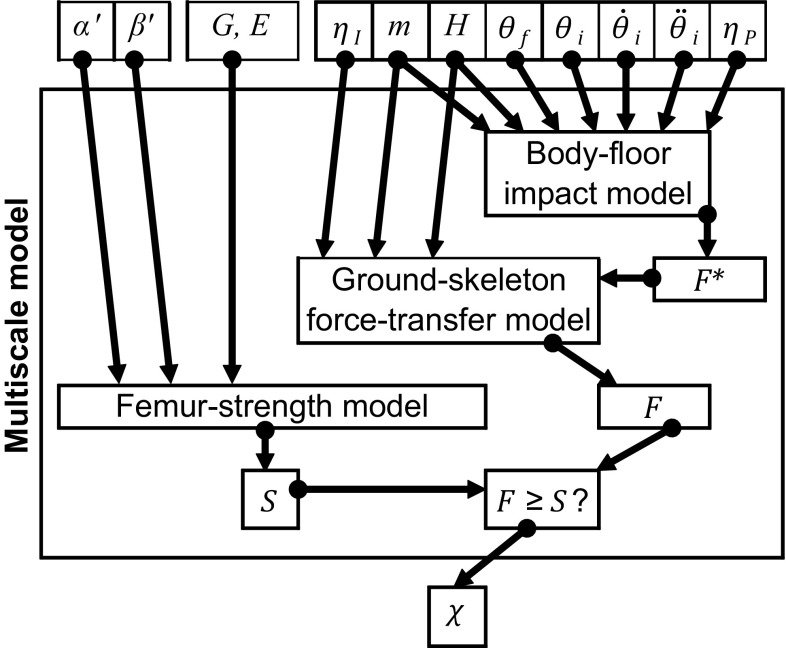
Orchestration of the multiscale model with input parameters measured at scales from whole body to organ (bone)

The fracture outcome of a specific fall is denoted by the binary variable *χ*. We set *χ* = 1 (fracture occurs) when a fall occurs with impact force magnitude exceeding bone strength (*F* ≥ *S*), and *χ* = 0 (fracture does not occur) otherwise. A fall is specified by the variables controlling the whole-body dynamics (*θ*_*i*_, *θ*_*f*_, $\dot{\theta }_{i} , \ddot{\theta }_{i}$), the postural and impact attenuation variables (*η*_*P*_, *η*_*I*_) and the impact orientation angles (*α*′, *β*′). *χ* also depends on the subject-specific properties of the femur and the body in which the femur is embedded. The femur is specified by its (discretized) geometry and elasticity properties (*G*, *E*), and the body is specified by its mass and height (*m*, *H*). Note that, the angles *α*′ and *β*′ depend on *α*, *β*, *ψ* (at impact), *G*, *H* and *θ*_*f*_. However, here *α*′ and *β*′ are considered as independent variables because *α*, *β* and *ψ* vary independently of *G*, *H* and *θ*_*f*_. As described below, *χ* is computed by using the ground–skeleton force-transfer model to bridge the models for body–floor impact and femur strength (Fig. 3). Given (*m*, *H*, *θ*_*i*_, *θ*_*f*_, $\dot{\theta }_{i} , \ddot{\theta }_{i}$, *η*_*P*_, *η*_*I*_), the fall-specific impact force *F* is obtained by sequentially executing the body–floor impact and the ground–skeleton force-transfer models. Given (*G*, *E*, *α*′, *β*′), the femur strength model is executed in parallel to obtain the fall-specific strength *S*. The determination of *χ* is complete as soon as *F* and *S* are known.

Next, *P* is determined by holding fixed the subject-specific variables (*m*, *H*, *G*, *E*) and accounting for the variability of fall-specific variables across falls. Let $p^{x}$ denote the probability density function (PDF) of any input variable *x* in the set (*α*′, *β*′, *θ*_*i*_, *θ*_*f*_, $\dot{\theta }_{i} , \ddot{\theta }_{i}$, *η*_*P*_, *η*_*I*_). Then the probability that a random fall will lead to a fracture is given by:

7 $$\begin{aligned}& P = \mathop \int \limits_{{\alpha^{\prime}}}^{{}} \mathop \int \limits_{ \ldots }^{{}} \mathop \int \limits_{{\eta_{I} }}^{{}} p^{{\alpha^{\prime}}} p^{{\beta^{\prime}}} p^{{\theta_{i} }} p^{{\theta_{f} }} p^{{\dot{\theta }_{i} }} p^{{\ddot{\theta }_{i} }} p^{{\eta_{P} }} p^{{\eta_{I} }}\\ &\quad \quad  \chi {\text{d}}\eta_{I} {\text{d}}\eta_{P} {\text{d}}\ddot{\theta }_{i} {\text{d}}\dot{\theta }_{i} {\text{d}}\theta_{f} {\text{d}}\theta_{i} {\text{d}}\beta^{\prime}{\text{d}}\alpha^{\prime}\end{aligned} $$

Admissible limits for each variable of integration specify the ranges of integration in Eq. (7). These limits are detailed in the next section, along with the PDFs $p^{x}$. *M* nominally uniformly spaced angle pairs (*α*′, *β*′) are considered within the limits of *α*′ and *β*′. *N* samples of the vector (*θ*_*i*_, *θ*_*f*_, $\dot{\theta }_{i} , \ddot{\theta }_{i}$, *η*_*P*_, *η*_*I*_) with PDFs $p^{{\theta_{i} }}$, …, $p^{{\eta_{I} }}$ are drawn using inverse-transformed Latin Hypercube (LH) sampling. Corresponding to the *M* pairs of (*α*′, *β*′) values and *N* samples of the (*θ*_*i*_, *θ*_*f*_, $\dot{\theta }_{i} , \ddot{\theta }_{i}$, *η*_*P*_, *η*_*I*_) vector, *M*×*N* values of *χ* are obtained by repeated executions of the first step described above. Monte Carlo (MC) integration method is applied over the (*θ*_*i*_, *θ*_*f*_, $\dot{\theta }_{i} , \ddot{\theta }_{i}$, *η*_*P*_, *η*_*I*_)-domain. Thus, the *M*×*N* values of *χ* are averaged *N* at a time, leading to *M* averaged-*χ* values. Finally, *P* is computed using numerical quadrature over the (*α*′, *β*′)-domain. Thus, averaged-*χ* values and PDFs *p*^*α′*^ and *p*^*β′*^ at any location in the (*α*′, *β*′)-domain are linearly interpolated using a triangular grid connecting the *M* discrete (*α*′, *β*′) points. Given a fall rate *n*, *ARF0* is known from Eq. (6) as soon as *P* is determined.

### ARF0 model input data

The subject-specific inputs—body mass *m* and height *H*, discretized geometry *G* and discretized elastic properties *E* of the proximal femur—were obtained from a retrospective cohort (validation cohort; Table 2) comprising 98 postmenopausal British women; the details of the cohort and of data acquisition are given in Qasim et al. (2016) and Yang et al. (2014). Briefly, one half of the cohort (fracture group) had been diagnosed with low-energy trauma fractures in the proximal femur; the other half (non-fracture group) were pair-matched for age, weight, and height with subjects in the fracture group. *G* and *E* are obtained using proximal femur CT image data. Distributions of body height, body mass and bone mineral density in the validation cohort reflect, by design, the distribution of osteopenia in the population referred to an osteoporosis specialist in a secondary care setting.

**Table 2:** Fixed, subject-specific and stochastic parameters of the multiscale model

Fixed parameters		
*c* = 0.554 (Croskey et al. 1922)	*∆t* = 0.09 s (Laing and Robinovitch 2008)	*n* = 0.65 (Gillespie et al. 2012)

Parameter (unit)	Fracture group (n = 49)	Non-fracture group (n = 49)
Subject-specific parameters: mean (SD) (Qasim et al. 2016; Yang et al. 2014)		
*m* (kg)	62.6 (14.3)	64.9 (12.1)
*H* (m)	1.58 (0.0653)	1.58 (0.0592)
Age (years)	75.4 (9.44)	74.7 (8.86)

Parameter (Unit)	Truncation values	Justifications and references
Fall stochasticity parameters		
*θ*_*i*_ (°)	*a* = 0	Falls from upright position (Robinovitch et al. 2013)
	*b* = 30	Minimum inclination from which fall recovery is not possible (Smeesters et al. 2001; Thelen et al. 1997; Wojcik et al. 1999).
*θ*_*f*_ (°)	*a* = 60	Falling on stairs with feet downward (Talbot et al. 2005); typical inclination from the vertical of stairs in the UK (HM Government 2013)
	*b* = 120	Falling with feet on chair and hip on ground (Talbot et al. 2005); seat height of standard chair: 44 cm (Wheeler et al. 1985); COM distance from feet for average subject: *h* = c*1.58 m = 87.5 cm; inclination of pendulum with horizontal = arcsin (44/87.5) ~ 30°
*cH*$\dot{\theta }_{i}$ (ms^−1^)	*a* = 0.00	Falls from a state of rest (Robinovitch et al. 2013)
	*b* = 1.40	Falls initiated with highest linear COM velocity achieved during level walk (Robinovitch et al. 2013; Terrier and Reynard 2015)
*cH*$\ddot{\theta }_{i}$ (ms^−2^)	*a* = 0.00	Falls initiated during unaccelerated and the most accelerated phases of gait (Hernandez et al. 2009)
	*b* = 5.10	
*η*_*P*_ (–)	*a* = 0.500	Based on dynamical models of falling (Sandler and Robinovitch 2001; van den Kroonenberg et al. 1995)
	*b* = 0.800	
*η*_*I*_ (–)	*a* = − 2.55	(1 − *a*) ≡ (1 − min *η*_*I*_^floor^)(1 − min *η*_*I*_^ext^)(1 − min *η*_*I*_^act^)
		(1 − *b*) ≡ (1 − max *η*_*I*_^floor^)(1 − max *η*_*I*_^ext^)(1 − max *η*_*I*_^act^)
		*η*_*I*_^floor^: 0–0.870, based on experiments of Laing and Robinovitch (2009) using synthetic hips without hip protectors (*η*_*I*_^act^ = 0, *η*_*I*_^ext^ = 0) impacting various flooring materials
	*b* = 0.914	*η*_*I*_^ext^: 0–0.338, based on experiments of Laing and Robinovitch (2008) considering synthetic hips impacting rigid floors (*η*_*I*_^act^ = 0, *η*_*I*_^floor^ = 0) with various hip protector designs
		*η*_*I*_^act^: –2.55–0, based on experiments of Robinovitch et al. (1991) conducted on rigid floors without hip protectors (*η*_*I*_^floor^ = 0, *η*_*I*_^ext^ = 0) with live subjects contracting or relaxing trunk and back muscles during fall

The fall parameters *θ*_*i*_, *θ*_*f*_, $\dot{\theta }_{i} , \ddot{\theta }_{i}$, *η*_*P*_, and *η*_*I*_ are described by normal distributions truncated symmetrically at ± 3 standard deviations (SDs) from the mean. Thus, the truncated distributions are fully specified by truncation points *a* and *b*, and the mean and SD of the non-truncated distribution are given by (*a* + *b*)/2 and (*b* – *a*)/6, respectively. Truncation points do not vary across subjects and are listed in Table 2.

Past studies have reported a strong correlation between trochanteric soft tissue thickness (*STT*) and *BMI* (Dufour et al. 2012; Schacter and Leslie 2014). For example in female subjects,

8 $$ STT\left( {cm} \right) \, = \, 0.23415*BMI(kg \, m^{ - 2} ) \, {-} \, 3.3444 $$

According to Eq. (8), a subject with a *BMI* of 14.3 possesses zero soft tissue thickness at the trochanter. The *BMI* range in the validation cohort is 14.4–36.4. Hence the predicted *STT* ranges from 0.0284–5.19 cm. Robinovitch et al. (1995) measured the impact between hard floors (*η*_*I*_^floor^ = 0) and cadaver pelvic regions (*η*_*I*_^act^ = 0) in the absence of hip protectors (*η*_*I*_^ext^ = 0). These conditions simplify Eq. (5) to *F* = (1 – *η*_*ST*_) F*. Robinovitch et al. (1995) reported the following dependence between *F* and *STT*

9 $$ F\left( {kN} \right) \, = \, 7.2 \, {-} \, 0.71*STT $$

In their experiments, F* was constant because Robinovitch et al. (1995) fixed the mass and the energy of the fall. Thus, for any *STT*, the ratio (7.2 − 0.71*STT)/(1 − *η*_*ST*_) is a constant. Defining *η*_*ST*_ = 0 as the attenuation when *STT* = 0, the constant above is found to be 7.2, which gives *η*_*ST*_ = 0.0986*STT for arbitrary *STT*. Using Eq. (8) it follows that:

10 $$ \eta_{ST} = \, 0.0231*BMI{-} \, 0.330 $$

The expected range of *η*_*ST*_ in the validation cohort is 0.00264–0.511. If a subject possesses a very small *BMI* such that *η*_*ST*_ is predicted to be negative using Eq. (10), it is reset to zero.

It was found that varying (*α*′, *β*′) in the domain [− 30°, + 30°] × [0, + 30°] resulted in the contact to initiate at points which, for all the subject-specific bone geometries analysed, covered nearly the entire greater trochanter surface. For a given fall, all (*α*′, *β*′) in the above range are assumed to have an equal probability of occurrence.

The fall rate *n* = 0.65 is considered fixed for all subjects and is close to the median value reported in the literature for the community-dwelling elderly population (Gillespie et al. 2012, see Appendix 8).

## Results

This section presents results from verification, uncertainty quantification and validation analyses of all four models detailed above. Verification relates to analysing the dependence of model predictions on numerical approximations made in model implementation. Uncertainty quantification relates to analysing the dependence of model predictions to measurement errors in model inputs. Validation relates to analysis of the differences between model predictions and clinical or experimental observation.

### Verification

The models for body–floor impact and for ground–skeleton force-transfer do not involve numerical approximations. Thereby, verification of these models is not required. In the femur strength model, numerical approximations arise due to FE discretization. Helgason et al. (2008) showed that for the mesh density used in the present model (average element edge length, 3.3 mm) the effect of further refinement leads to less than 1% change in predicted strains. The predicted strength, which depends on the predicted strains, is therefore independent of the FE mesh. Hence, the femur strength model is also considered verified.

For the multiscale model, the only numerical approximation made is in the computation of *P* in Eq. (7). In particular, MC integration over the variables *θ*_*i*_, *θ*_*f*_, $\dot{\theta }_{i} , \ddot{\theta }_{i}$, *η*_*P*_, and *η*_*I*_ and numerical quadrature over the variables *α*′ and *β*′ require verification. This is performed in three steps. In the first step, samples of the vector (*θ*_*i*_, *θ*_*f*_, $\dot{\theta }_{i} , \ddot{\theta }_{i}$, *η*_*P*_, *η*_*I*_) of different sizes (*N*) are drawn using inverse-transformed LH sampling as previously described. Estimates of mean and variance of *x* in dependence of sample size *N* are obtained, where *x* is any variable in (*θ*_*i*_, *θ*_*f*_, $\dot{\theta }_{i} , \ddot{\theta }_{i}$, *η*_*P*_, *η*_*I*_). This establishes a nominally verified LH sample size. In the second step, this nominally verified LH sample size is held fixed for the integration in Eq. (7) over the (*θ*_*i*_, *θ*_*f*_, $\dot{\theta }_{i} , \ddot{\theta }_{i}$, *η*_*P*_, *η*_*I*_) domain. The integration in Eq. (7) over the (*α*′, *β*′) domain is carried out for different numbers (*M*) of discrete orientation pairs. The dependence of *ARF0* on *M* is determined, and the number of orientation pairs needed to ensure that a verified numerical quadrature is obtained. In the third step, keeping the number of orientation pairs fixed, the integral in Eq. (7) is computed for different LH sample sizes *N*, thus verifying the MC integration.

**Fig. 4:**
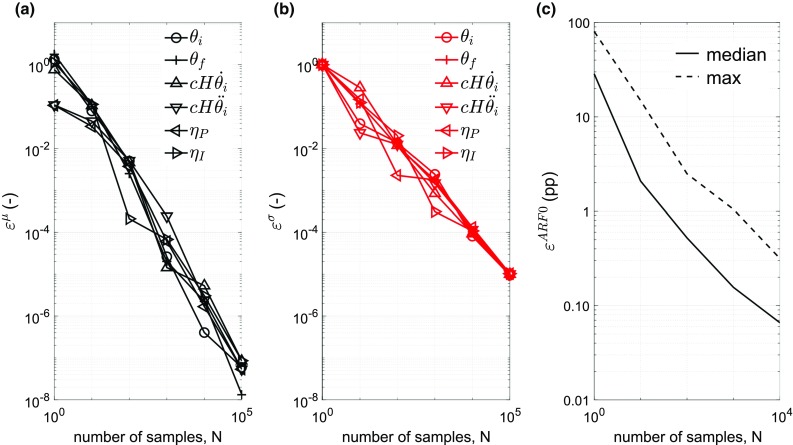
Dependence of *ARF0* on Latin Hypercube sample size. Normalized errors in (**a**) sample mean and **b** sample variance of input parameters of multiscale model; **c** median and maximum absolute error in model prediction *ARF0* (expressed as percentage points, pp) over the validation cohort. For the input parameters, errors are defined with respect to the theoretical mean and variance values of the truncated normal distribution. For the model output, errors are defined with respect to the *ARF0* values for the largest sample size *N = *10^5^. Errors in model output for *N = *10^5^ are zero by definition and hence omitted

For the first step, the errors in the mean and variance of finite LH samples are quantified by *ε*^*μ*^ = |μ* − *μ*|/√*σ*^2^ and *ε*^*σ*^ = |(σ*)^2^ − *σ*^2^|/*σ*^2^. Here, *μ* and *σ*^2^ are the theoretical mean and variance of the truncated normal distribution for each input, and the symbols with asterisk denote corresponding estimates based on the finite sample (size *N*). Figure 4a, b shows that *ε*^*μ*^, *ε*^*σ*^ < 0.001 for *N* ≥ 10^4^. This verifies LH sampling on each variable individually.

For the second step, we consider subject-specific bone strength at *M* discrete orientations in the (*α*′, *β*′) domain. The greatest difference in bone strength between any two orientations is a measure of variability in strength in the subject. This variability is found to be the highest for subject #50 of the validation cohort. Thus, it is expected that the error in evaluating the integrals over the (*α*′, *β*′) domain in Eq. (7) using numerical quadrature will be largest for the ‘worst-case scenario’ of subject #50. It is found that *ARF0* = 31.0% for subject #50, when using *N* = 10^4^ for MC integration and *M* = 231. In comparison, *ARF0* is modified by 3.54 pp, 2.32 pp, 1.92 pp and 0.0503 pp when *M* = 4, 15, 33, and 66 respectively. Thus, numerical quadrature over the (*α*′, *β*′) domain is verified to a tolerance of 1.92 pp when using *M* = 33.

In the third step, *ε*^*ARF0*^ (*i*, *N*) = |*ARF0* (*i*, *N*) − *ARF0* (*i*, 10^5^)| is used to define the error in estimating *ARF0* for subject *i* using Eq. (7) with LH sample size *N*, compared to with LH sample size *N* = 10^5^. Figure 4c shows that as *N* increases, both the median and maximum (taken over the validation cohort) of *ε*^*ARF0*^ decrease; becoming negligibly small (0.0658 pp and 0.316 pp, respectively) for *N* = 10^4^ when compared to the minimum, median and maximum values of *ARF0* (1.93, 36.1 and 81.6%, respectively) computed using *N* = 10^5^.

In summary, *ARF0* can be determined to a numerical precision of 1.92 pp when using *N* = 10^4^ and *M* = 33.

### Uncertainty quantification

The uncertainty of the body–floor impact model prediction F* for any input vector ***x*** = (*m*, *H*, *θ*_*i*_, *θ*_*f*_, $\dot{\theta }_{i} , \ddot{\theta }_{i}$ and *η*_*P*_) is (Fornasini 2008)

11 $$ s_{{F^{*} }} = \sqrt {\mathop \sum \limits_{x}^{{}} s_{x}^{2} \left( {\frac{{\partial F^{*} }}{\partial x}} \right)^{2} } $$

where *x* is an element of ***x***, $s_{x}^{2}$ is the uncertainty (variance) in the measurement of *x* and the partial derivative is evaluated at the point of the input parameter domain where the uncertainty $s_{{F^{*} }}$ is to be computed. In the following, instead of using Eq. (11), the approximation

12 $$ \tilde{s}_{{F^{*} }} = \sqrt {\left( {\mathop \sum \limits_{X}^{{}} s_{X}^{2} } \right)\left( {\frac{{F^{*}_{ \hbox{max} } - F^{*}_{ \hbox{min} } }}{{\left| {\varvec{X}_{ \hbox{max} } - \varvec{X}_{ \hbox{min} } } \right|}}} \right)^{2} } $$

is used, where ***X*** is a location in the domain of input variables to which F* is highly sensitive (see below), *X* is an element of ***X***, 〈F*〉 denotes average of F* taken at ***X*** by varying the remaining inputs over their full ranges, 〈F*〉_max_ and 〈F*〉_min_ are the extreme values of 〈F*〉 over all ***X***, and ***X***_max_ and ***X***_min_ are respectively the locations where these extrema occur. Thus, $\tilde{s}_{{F^{*} }}$ provides a location-independent quantification of uncertainty. To determine the input variables to which F* is highly sensitive, the global first-order sensitivity indices *S*_*x*_ are computed for each input variable *x* (Sobol 2001). The variables for which *S*_*x*_ (ordered from largest to smallest) sum to just over 80% are chosen as the ones to which F* is highly sensitive. Following Saltelli et al. (2010), *S*_*x*_ are determined by computing the impact force magnitude F* for samples of input parameters drawn from uniform distributions in the following ranges: *m*, 31.0–101 kg; *H*, 1.45–1.73 m; and for the remaining parameters, in the ranges given by truncation values (Table 2). By using a sample size of *N* = 10^5^, it is ensured that sensitivity indices (Table 3) are determined correct to 0.670 pp. It is found that F* is highly sensitive only to *m* and *θ*_*f*_. From literature sources, measurement uncertainties in *m* and *θ*_*f*_ are found to be *s*_*m*_ = 4.10 kg (inter-observer error, Ulijaszek and Kerr 1999) and $s_{{\theta_{f} }}$ = 3.70° (inter-examiner error, Della Croce et al. 2005), respectively. The extreme average values 〈F*〉_max_ = 3650 N and 〈F*〉_min_ = 809 N are computed by binning the sample of F* (used to compute the sensitivity indices) over a 10×10 regular grid in the domain of *m* and *θ*_*f*_. These correspond to ***X***_max_ = (*m*, *θ*_*f*_)_max_ = (101 kg, 30°) and ***X***_min_ = (*m*, *θ*_*f*_)_min_ = (31.0 kg, − 30°). Using Eq. (12), the uncertainty in the prediction of F* is then found to be $\tilde{s}_{{F^{*} }}$ = 166 N.

**Table 3:** First-order sensitivity indices (*S*_*x*_) of F* (body–floor impact model), *F* (ground–skeleton force-transfer model) and *ARF0* (multiscale model) to various model input. Key: *m*, body mass; *θ*_*f*_, final angle of fall; *η*_*P*_, postural attenuation coefficient; *H*, body height; *η*_*I*_, impact attenuation coefficient; 〈*S*〉, bone strength averaged over all impact orientations

	*m*	*θ*_*f*_	*η*_*P*_
F*			
*** S***_***x (%)***_	66.0	14.6	10.6

	*m*	*H*	*η*_*I*_
***F***			
*** S***_***x (%)***_	17.1	2.39	75.7

F*	*m*	*H*	〈*S*〉
***ARF0***			
*** S***_***x (%)***_	1.06	0.220	84.1

All sensitivity indices are based on sample sizes of 10^5^

The uncertainty of the ground–skeleton force-transfer model was evaluated in a similar manner, with *m*, *H*, *η*_*I*_ and F* as the input variables. As F* is not independent of *m* and *H*, an LH sampler is used to draw independent samples of *m*, *H*, *θ*_*i*_, *θ*_*f*_, $\dot{\theta }_{i} , \ddot{\theta }_{i}$ and *η*_*P*_ as above, and of *η*_*I*_ from a uniform distribution given by its truncation values (Table 2). *S*_*x*_ are obtained corresponding to *m*, *H* and *η*_*I*_ (Table 3) which converge for samples of size *N* = 10^5^, beyond which sensitivity indices change by less than 0.130 pp. Based on the 80% threshold for sum of first-order indices, it is found that *F* is highly sensitive to *m* and *η*_*I*_. Literature sources give the measurement uncertainties in *m* and *η*_*I*_ as *s*_*m*_ = 4.10 kg and $s_{{\eta_{I} }}$ = 0.342 (Hurkmans et al. 2003), respectively. 〈*F*〉_max_ = 5040 N, 〈*F*〉_min_ = 277 N, ***X***_max_ = (*m*, *η*_*I*_)_max_ = (66.0 kg, − 2.55) and ***X***_min_ = (*m*, *η*_*I*_)_min_ = (31.0 kg, 0.568) are computed by binning the sample of *F* over a 10 × 10 regular grid in the domain of *m* and *η*_*I*_. Using Eq. (12), the uncertainty in the prediction of *F* is found to be $\tilde{s}_{F}$ = 558 N.

The uncertainty in determining femur strength *S* depends on the variable to which bone strength is more sensitive (bone geometry *G* or bone elasticity *E*) over the range of variation of these variables in the population. To the best of our knowledge, there is no study that has developed a parameterization for *G* and *E* which satisfactorily captures the variation in the elderly female population. Here, we consider DXA–aBMD as a surrogate measure of the volumetric bone density (which in turn determines bone elasticity *E*) and body height *H* as a surrogate measure of bone geometry *G*. For the subjects in the validation cohort, the variations in the minimum and maximum subject-specific bone strengths, i.e. respectively $\mathop {\hbox{min} }\nolimits_{{\left( {\alpha^{\prime},\beta^{\prime}} \right)}} S\left( {\alpha^{\prime},\beta^{\prime}} \right)$ and $\mathop {\hbox{max} }\nolimits_{{\left( {\alpha^{\prime},\beta^{\prime}} \right)}} S\left( {\alpha^{\prime},\beta^{\prime}} \right)$, are explained up to 24.8% and 42.3% by the variations in DXA-aBMD, but only up to 7.38% and 8.14% by variation in body height *H*. The level of explanatory power of DXA–aBMD in relation to bone strength is similar to that reported elsewhere (Muehleman et al. 2000) and is expectedly higher than that of body height. Hence, we only consider the uncertainty in predicting *S* due to uncertainties in measuring *E*. (Qasim et al. 2016) reported that uncertainties in determining *E*, due to using three different tube-current levels (100, 150 and 200 mA) when scanning the off-line phantom, resulted in femur strength uncertainties below 3%. In the validation cohort, this uncertainty is the largest ($\tilde{s}_{S}$ = 190 N) for the maximum predicted strength of 6329 N.

**Fig. 5:**
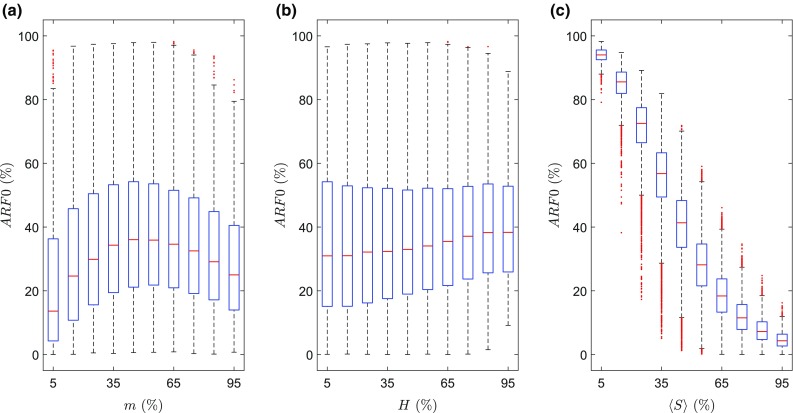
The variation of *ARF0* in a virtual population of 10^5^ subjects in dependence of subject-specific parameters: **a** body mass, *m*; **b** body height, *H*; and **c** bone strength averaged over all orientations, $S = \mathop {\text{mean}}\limits_{{\left( {\alpha^{\prime},\beta^{\prime}} \right)}} S\left( {\alpha^{\prime},\beta^{\prime}} \right)$. Percentage values on the horizontal axes are with respect to the range of the corresponding parameter. In each box, the red horizontal line denotes the median value, the top and bottom edges of the box denote the 25th and 75th percentiles, whiskers dots denote values at 1.5 times the interquartile range beyond the box edges and red dots denote outliers

The uncertainty of *ARF0* is determined with respect to the uncertainty in *m*, *H* and *S*. In the validation cohort, *m* and *H* are normally distributed (Table 2). In order to numerically evaluate the sensitivity of *ARF0*, a parameterization is required that captures the variation of bone strength in the elderly female population as represented by the validation cohort. In this cohort, strength values are normally distributed at 26 of the 33 orientations (Anderson–Darling test, *p* = 0.05). Hence, mean and standard deviation spatial distributions of bone strength offer a potential parameterization. This is confirmed by the fact that in 86 of the 98 subjects, the spatial distribution of strength for subject *j*, denoted $S^{j} \left( {\alpha^{\prime},\beta^{\prime}} \right)$, is at least moderately correlated (coefficient of correlation, *r* ≥ 0.5) with the strength distribution averaged over subjects $\mathop {\text{mean}}\nolimits_{j} S^{j} \left( {\alpha^{\prime},\beta^{\prime}} \right)$. It is also found that in the validation cohort, distributions of body mass *m*, body height *H* and strength averaged over all orientations $S = \mathop {\text{mean}}\nolimits_{{\left( {\alpha^{\prime},\beta^{\prime}} \right)}} S^{j} \left( {\alpha^{\prime},\beta^{\prime}} \right)$ are weakly, but non-negligibly, correlated: *r* (*m*, *H*) = 0.429, *r* (*m*, 〈*S*〉) = 0.264 and *r* (*H*, 〈*S*〉) = 0.294. Hence, the synthesized Fourier amplitude sensitivity testing (SFAST) method (Xu and Gertner 2008) was applied to obtain first-order sensitivity indices (also denoted *S*_*x*_) of *ARF0*. The variables *m*, *H* and *S*(*α*′, *β*′) are sampled from normal distributions with the mean and SD identical to those of the validation cohort, truncated symmetrically at ± 3SD; the samples possess the correlations mentioned above (Iman and Conover 1982). For populations with *N* ≥ 10^5^ individuals, SFAST-computed *S*_*x*_ change by less than 0.0209 pp; converged indices are reported in Table 3. It is found that *ARF0* is highly sensitive to *S* only (again based on the 80% threshold), seen in Fig. 5 as the much smaller variation in *ARF0* within subjects possessing a fixed bone strength than the variation in *ARF0* within subjects possessing a fixed body mass or a fixed body height. Uncertainties in determining *S* were found to be less than $\tilde{s}_{S}$ = 190 N above. 〈*ARF0*〉_max_ = 93.8%, 〈*ARF0*〉_min_ = 4.83%, ***X***_max_ = (*S*)_max_ = 393 N and ***X***_min_ = (*S*)_min_ = 4420 N are computed by binning the sample of *ARF0* over a 10 regularly spaced points in the range of *S*. Using Eq. (12) the uncertainty in the prediction of *ARF0* is found to be $\tilde{s}_{ARF0}$ = 4.00 pp.

### Validation

Hip impact velocity predicted by the body–floor impact model averaged 2.82 m s^−1^ (SD 0.335 m s^−1^) which compares with 3.01 m s^−1^ (SD 0.83 m s^−1^) (Feldman and Robinovitch 2007), 2.75 m s^−1^ (SD 0.42 m s^−1^) (van den Kroonenberg et al. 1996) and 1.16–2.73 m s^−1^ (predicted, Lo and Ashton-Miller 2008) in previous studies. The output *F* of the ground–skeleton force-transfer model averages 2.66 kN (SD 0.925 kN) which compares with 0.475–2.5 kN (Laing and Robinovitch 2009) and 1.23–5.57 kN (predicted, Lo and Ashton-Miller 2008) reported previously. For the femur strength model, Schileo et al. (2014) used a similar FE modelling methodology to predict the strength of cadaveric bones and compared these with experimentally measured strength. The standard error of estimate of the FE predicted bone strengths was found to be 15% of the average measured strength value. When the minimum bone strength across all 33 distinct orientations in each subject was considered, it was found to classify the fracture and non-fracture subjects with an area under the receiver operating characteristic (ROC) curve (AUC) was found to be 0.82 (Viceconti et al. 2018).

**Fig. 6:**
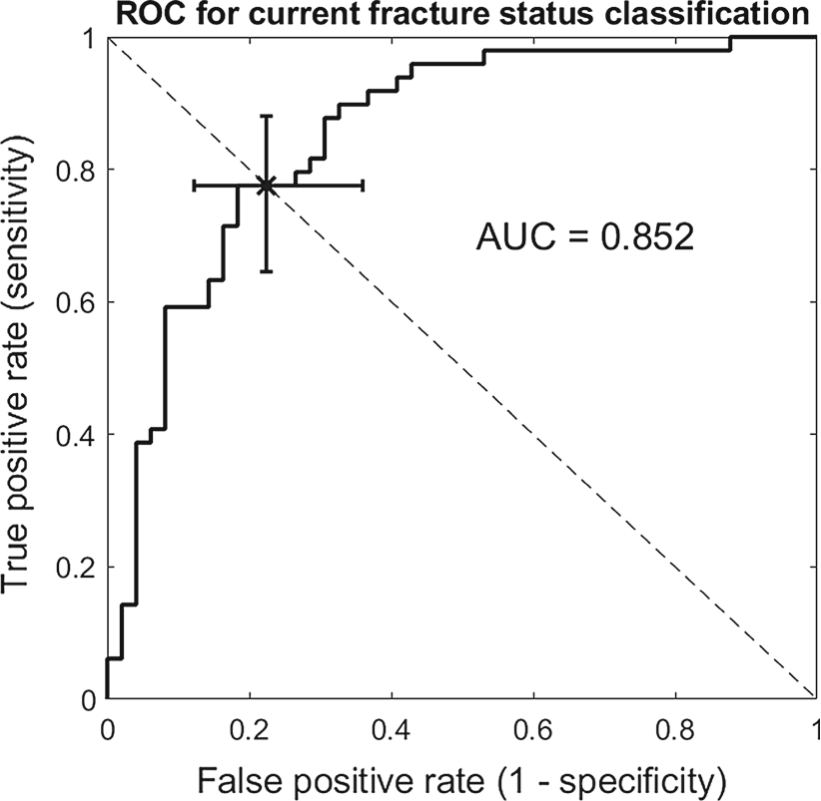
Receiver operating characteristic (ROC) curve for the classification of current fracture status in the postmenopausal cohort using *ARF0*. The cross corresponds to *ARF0 = *37.4% where specificity is 77.6% and sensitivity is 81.6% (error bars denote the respective 95% confidence intervals). AUC refers to area under the ROC curve

The multiscale model was validated as follows. A Mann–Whitney test falsified the null hypothesis that the average *ARF0* for the fracture group (48.4%) was equal to the average *ARF0* for the non-fracture groups (24.6%) up to a significance level of 0.001. A Hosemer–Lemeshow test showed no evidence of poor fit (*p* = 0.328) when using a univariate logistic regression model to predict current fracture status based on *ARF0*. The ROC curve analysis (Fig. 6) shows that the most optimal classification at the *ARF0* = 37.4% threshold, with 77.6% specificity (95% CI: 63.4%–86.5%) and 81.6% sensitivity (95% CI: 68.3%–91.1%). The area under the ROC curve AUC = 0.852 (95% CI, 0.753–0.918) was significantly higher for *ARF0* when compared to AUC = 0.750 corresponding to the standard-of-care predictor which is the DXA-based T-score at the femoral neck (Qasim et al. 2016), and also when compared to AUC = 0.82 corresponding to the CT-FE based minimum bone strength predictor (Viceconti et al. 2018). The classification by *ARF0* was found to be significant after adjusting for femoral neck T-score (*p* < 0.001).

## Discussion

The multiscale prediction *ARF0* described here was verified with respect to all numerical approximations and achieved an overall error tolerance (1.92 pp). An additional uncertainty of 4.00 pp in predicted *ARF0* was ascribed to the uncertainty in determining bone strength. This was due to high sensitivity of *ARF0* to bone strength, which is in line with the strong dependence of fracture risk indices on DXA-aBMD (Bouxsein et al. 2007; Sarvi and Luo 2015) and the excellent classification of current fracture status using CT-FE based bone strength measures (Qasim et al. 2016). Nevertheless, errors in *ARF0* due to numerical approximations and propagated uncertainties are much smaller than the median *ARF0* (36.1%) in the cohort. Hence, differences in predicted *ARF0* between two typical subjects in the cohort are expected to remain statistically significant even in the presence of these errors.

Previous mechanistic models that predicted fracture risk based on fall dynamics and bone strength (Bouxsein et al. 2007; Sarvi and Luo 2015) reported an association between the fracture risk predictor and fracture status. In comparison, *ARF0* is not only found to be associated with fracture status, but is found to additionally classify fracture status with satisfactory sensitivity and specificity. This is possibly because *ARF0* includes the variability in hip impact forces across falls in the same subject, which was not captured in Bouxsein et al. (2007) and Sarvi and Luo (2015). The classification of fracture status, compared head-to-head on the same cohort, is significantly higher using *ARF0* (AUC = 0.852) than using bone strength alone (AUC = 0.82) or using DXA-aBMD alone (AUC = 0.75) (Qasim et al. 2016; Viceconti et al. 2018). This additional predictive power is made possible by including subject-specific fall dynamics and hip impact mechanics, which are excluded from mechanistic models based only on bone quality (Adams et al. 2018; Keyak et al. 2011; Panyasantisuk et al. 2018; Qasim et al. 2016). Thus, the main new insight from the present study is that fracture status in postmenopausal women is determined by the competition between the impact force (applied at the hip during a fall to the side) and the strength of the femur (under a side-fall loading condition) and the variability in impact force across potential falls. Note that it is not possible gain such ‘mechanistic’ insights from models employing statistical regression for fracture risk predictions (Hippisley-Cox and Coupland 2009; Kanis et al. 2008).

For the component models, predicted velocities of approach at the instant of fall and peak impact force transferred to the skeleton were found to agree excellently with a large number of experimental studies. The uncertainties in determining impact force on the body (166 N) were much smaller than the standard deviation of its entire variation (770 N). This uncertainty is significantly influenced by the uncertainties in the measurements of body mass and final angle of fall, which may be considered acceptable for the purpose of determining the impact force on the body. The relatively small sensitivity of impact force on the body to the parameter corresponding to postural attenuation implies that reduced-order models (of lower accuracy) of postural attenuation can be applied without significantly affecting the accuracy of impact force prediction. The uncertainty in determining hip impact force (558 N) was not much smaller than the standard deviation of its entire variation (925 N). This uncertainty depended most strongly on the uncertainty in the measurements of impact attenuation coefficient. This underlines the importance of developing better models to account for the role of agents such as flooring materials, muscle activation state and hip protectors.

The fall dynamics model and the ground–skeleton force-transfer model, leading up to the determination of attenuated hip impact force magnitude *F* in Eq. (5), are substantial simplifications of past model development in this area (Laing and Robinovitch 2008, 2009; Lo and Ashton-Miller 2008; Robinovitch et al. 1991, 1995; Sandler and Robinovitch 2001; van den Kroonenberg et al. 1995). The model reductions made here reflect the fact that the objective of the present models was simpler, which was to determine the variation of peak attenuated impact force magnitude across falls. Thus, it was justified to omit those features in the present model that are required only to predict quantities such as the motions and torques at lower limb joints, the motion of and the impact at upper extremities, the rotation of the body about its own axis, the transient response of the pelvis–femur joint. The model also omits those features that are required only to predict peak impact force magnitude in a fall-specific manner. However, the present model includes features such as postural attenuation coefficient *η*_*P*_ that determine the variability in peak impact force magnitude across falls in the same subject; features such as body mass *m* that determine the subject-specificity of peak impact force magnitudes; and features such as proportionality factor *k* and impact duration *∆t* that determine the complexity of fall but are constant across subjects and falls. This model reduction is novel to the best of our knowledge.

Differences between the FE modelling pipeline used here and the approaches used by Bessho et al. (2007), Keyak et al. (1997, 2005) and Nishiyama et al. (2014) have been discussed extensively in Falcinelli et al. (2014), Qasim et al. (2016) and Schileo et al. (2014), where the FE modelling pipeline used was identical to that in the present paper. Briefly, the material model (stress–strain relationship) used in our pipeline is linear elastic, while a nonlinear model was used by Bessho et al. (2004, 2007, 2009). The FE models used by Keyak (2001), Keyak et al. (2005), Keyak and Rossi (2000), Keyak et al. (1997, 2011) and Nishiyama et al. (2014) use voxel meshes and the failure load is computed based on strains throughout the volume of the bone, as opposed to tetrahedral meshes used in the present modelling pipeline and failure load defined by strains on the surface of the femur. In the present modelling approach, the anisotropy at the organ scale is captured by allowing the elastic modulus to vary element-wise. This has been shown to be adequate in predicting failure load in cadaveric femurs accurately under various loading conditions Schileo et al. (2014). Falcinelli et al. (2014) and Qasim et al. (2016) showed that the same modelling pipeline as Schileo et al. (2014) and including only organ scale anisotropy yields bone strength values under different loading conditions in living subjects which classify fracture status in these subjects with high accuracy.

Our study has several limitations. In its guideline for clinical assessment of fracture risk, NICE (2013) assumes that 3% of all falls in the elderly lead to fracture. Although the basis for this estimate is not clear to the authors, for an annual fall rate of 0.65, this estimate leads to an *ARF0* of 1.96% (= 1 – (1 −  0.03)^0.65^). This is much smaller than the median *ARF0* of 36.1% in our entire cohort. Our cohort was drawn from a population of elderly women with osteopenia who are referred to an osteoporosis specialist in a secondary care setting. As such, the distribution of bone strength in this population is expected to be significantly lower than that in the general population considered in the NICE (2013) report. As bone strength predicts nearly 84% of the variation in *ARF0*, it is expected that for the general population the multiscale model will predict a much lower average *ARF0* consistent with the findings of the NICE (2013) report. Indeed, this is indicated by Fig. 5c where the median *ARF0* at 95% of the strength range is found to be 4.32%. It would of course be interesting to directly perform the computational prediction of *ARF0* in the general population, but that is outside the scope of the current work. Moreover, by design, half (50%) of the cohort had sustained a hip fracture. Thus, it is expected that the population represented by the cohort will possess ARF0 close to 50%.

Fall risk is expected to vary from one subject to another. Thus, ideally, a measurement of some subject-specific quantifier of fall risk is needed. Such a quantifier could not be identified among the variables measured on the validation cohort considered in this study. Fall rate is an accepted measure of fall risk that is often reported at the population level in observational studies and is readily interpreted within the frequentist definition of *ARF0*. Thus all subjects in our validation cohort were assumed to possess the same fall rate observed in a population similar to the cohort (Gillespie et al. 2012). Nevertheless, the lack of a subject-specific measure of fall risk could have influenced the results.

Soft tissue attenuation (*η*_*ST*_) directly determines peak impact force at the hip. Hence the lack of an accepted standard for measuring *η*_*ST*_ in vivo may affect the determination of peak impact force magnitudes and thereby affect the prediction of *ARF0*. We developed a regression model for *η*_*ST*_ based on soft tissue thickness (STT) using the experimental data of Robinovitch et al. (1995) and used a regression model to determine STT from body mass index (BMI) (Dufour et al. 2012). The sources of error in determining *η*_*ST*_ in this manner are: errors in the regression models and uncertainties in clinical measurements of body mass (4.10 kg, Ulijaszek and Kerr 1999) and body height (0.0140 m, DiMaria-Ghalili 2006). The coefficients of determination and the variances of the outcome variables of the regression models reported in past studies provide the error estimates of the regression models (Bouxsein et al. 2007; Robinovitch et al. 1995). These known sources of measurement uncertainties lead to a net 13.2% uncertainty in the predicted value of *η*_*ST*_. Being much smaller than the coefficient of variation of *η*_*ST*_ across the cohort (43.5%), the influence of these sources of uncertainties on *ARF0* is somewhat limited. The evidence in the literature regarding the role of soft tissue attenuation in determining fracture risk is also inconclusive (Compston et al. 2011; De Laet et al. 2005). Note that we considered STT only at the greater trochanter, similar to past studies (Dufour et al. 2012; Nielson et al. 2009; Schacter and Leslie 2014) and did not include the heterogeneity of STT within the hip region. To the best of our knowledge, there is no quantification of this heterogeneity in the literature, and its effect on the predicted *ARF0* merits further investigation.

The probability distributions of fall parameters were considered fixed in the present study. In doing so, the epistemic uncertainty therein is ignored. It is likely that the distribution(s) are modified by disease or intervention. Thus, currently the model cannot be used to investigate ‘what-if’ scenarios, such as how *ARF0* distribution in a population is modified in response to a disease or an intervention. Further empirical studies that can close the epistemic uncertainties in the fall parameters are needed to investigate such ‘what-if’ scenarios.

Another related shortcoming of the present model is the assumption of independence between various parameter distributions. This is also an area that requires careful empirical research to clarify epistemic uncertainties. One particularly weak assumption is that the fall energy attenuation parameter accounting for postural defence *η*_*P*_ is independent of the impact force attenuation parameter accounting for muscle activation *η*_*I*_^act^, as both attenuation mechanisms depend heavily on the activation of muscles in the lower limbs. It is likely that as muscle activation increases, *η*_*P*_ increases while *η*_*I*_^act^ decreases—a dependence that was not included in the present study due to lack of quantitative information.

The definition of *ARF0* in the present paper can readily be extended to *ARF10*, the absolute risk of fracture over a 10-year period, a measure of risk that is more prevalent in the clinical setting (Kanis et al. 2009). This would require accounting for, within a 10-year period, (a) a higher number of falls, (b) changes to fall severity and fall impact parameters due to ageing and (c) loss of bone mineral density due to remodelling. The methodological framework presented here is currently being extended to enable *ARF10* prediction.

## Conclusion

In this study, a multiscale model was developed to predict the current absolute risk of fracture *ARF0*. The model accounted for fall rate, stochasticity of fall scenarios including fall kinematics, postural reflex and fall impact attenuation conditions, and bone organ geometry and elasticity. The predictions of the multiscale model and its component models were verified to be independent of the numerical approximations made therein. In particular, it was found that *ARF0* can be determined using the model with an error much smaller than its variation across subjects. Uncertainties in the predictions of the multiscale model and its component models were quantified in dependence of uncertainties in the measurement of model inputs. In particular, it was found that predicted *ARF0* possessed an uncertainty that was mainly dependent on the uncertainty in the determination of bone strength, but was also much smaller than inter-subject variation. Predictions of multiscale model and its component models were validated against experimental and clinical observations. Specifically, predicted *ARF0* could classify the current fracture status of subjects in a postmenopausal cohort with high accuracy, sensitivity and specificity. In a head-to-head comparison on the same cohort, the accuracy of classifying current fracture status using *ARF0* was found to be significantly higher than predictors representing the standard-of-care (DXA-aBMD) and the state-of-the-art (based on CT-FE bone strength only). In conclusion, the *ARF0* model developed in this study provides a validated mechanistic explanation for fracture risk in dependence of fall severity and bone strength.
